# Occupational disparities in bladder cancer survival: A population‐based cancer registry study in Japan

**DOI:** 10.1002/cam4.2768

**Published:** 2019-12-11

**Authors:** Masayoshi Zaitsu, Hye‐Eun Lee, Sangchul Lee, Takumi Takeuchi, Yasuki Kobayashi, Ichiro Kawachi

**Affiliations:** ^1^ Department of Public Health Graduate School of Medicine The University of Tokyo Tokyo Japan; ^2^ Department of Social and Behavioral Sciences Harvard T.H. Chan School of Public Health Boston MA USA; ^3^ Korea Institute of Labor Safety and Health Seoul Republic of Korea; ^4^ Department of Urology Seoul National University Bundang Hospital Gyunggi‐do Republic of Korea; ^5^ Massachusetts General Hospital Cancer Center Boston MA USA; ^6^ Department of Urology Kanto Rosai Hospital Kawasaki Japan

**Keywords:** bladder cancer, Japan, occupation, population‐based, socioeconomic status, survival

## Abstract

**Background:**

Little is known about occupational disparities in bladder cancer survival.

**Methods:**

Using data from a population‐based cancer registry (1970‐2016), we identified 3593 patients with incident bladder cancer diagnosed during 1970‐2011 who completed occupational information. The patients were followed for 5 years (median follow‐up time 5.0 years). Their longest‐held occupations at incident bladder cancer diagnosis were classified according to a national standardized classification. Hazard ratios (HRs) and 95% confidence intervals (CIs) for overall death were estimated by Cox proportional hazard model, adjusted for age, sex, and year of diagnosis. Clerical workers served as the reference group.

**Results:**

Overall prognosis was fair in this population (5‐year overall survival, 61.9%). Compared with patients in clerical jobs, survival was poorer for those in professional and managerial jobs (mortality HR 1.36; 95% CI 1.09‐1.69), sales and service jobs (HR 1.25, 95% CI 1.01‐1.56), construction jobs (HR 1.83, 95% CI 1.40‐2.38), and manufacturing jobs (HR 1.32, 95% CI 1.05‐1.66), as well as those not actively employed (HR 1.27, 95% CI 1.02‐1.58). A similar pattern was observed in the subgroup analyses restricted to male patients as well as additional analyses adjusted for potential prognostic variables (eg, stage) with multiple imputation.

**Conclusion:**

We documented occupational disparities in bladder cancer survival in Japan. However, the pattern of disparity did not favor highest occupational groups.

## Graphical abstract

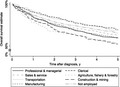

## 1 INTRODUCTION

Bladder cancer, which is four times more common in men compared with women, is the ninth most common cancer worldwide, and in 2012, male and female age‐standardized incidence rates were, respectively, 9.6 and 2.2 per 100 000 population in Japan.^1^ Although bladder cancer incidence in Japan is lower compared with that in Western countries, it is the highest in the East‐Asian region.^1^

The two most common risk factors for bladder cancer are smoking and occupational exposures to carcinogens.^2^ Smoking approximately quadruples the risk, and 50% of new bladder cancer cases are attributable to smoking.^3^ Occupational exposure to carcinogens (specifically, aromatic amines) is known to increase bladder cancer risk in occupations such as dye‐making, tobacco, rubber, and leather workers, printers, and hairdressers.^2^, ^4^, ^5^, ^6^ In Japan, an epidemic of bladder cancer incidence caused by exposure to ortho‐toluidine was reported in dye‐making workers,^7^ and ortho‐toluidine‐related bladder cancer has been designated as an occupational disease. Fortunately, occupational regulations have reduced this source of exposure in most countries.^2^

However, occupational differences in bladder cancer *survival* remain sparsely documented. In the Western context, several studies suggested that bladder cancer patients from blue‐collar job backgrounds (eg, manufacturing and mining) had a worse prognosis compared with white‐collar counterparts (eg, professionals and managerial workers).^8^, ^9^, ^10^ Clinical and pathological features (eg, pathology, stage, and treatment), as well as smoking behavior and environmental factors, are thought to underlie this monotonic pattern of occupational gradient in survival: that is, higher occupational class workers enjoy more favorable bladder cancer survival.^6^, ^8^, ^9^, ^10^ Yet, to the best of our knowledge, no studies have evaluated occupational disparities in bladder cancer survival in the non‐Western setting. In addition, in Japan, working in managerial and professional positions, the highest occupational class background, may not guarantee the best health outcome in all‐cause and cancer‐specific mortality and cardiovascular risks,^11^, ^12^, ^13^ which contrasts with the monotonic occupational gradient widely seen in the Western setting.

Accordingly, the goal of this study was to elucidate the association between occupation and bladder cancer survival in Japan. Using a population‐based cancer registry data set of bladder cancer, we primarily examined whether occupational disparities exist in bladder cancer survival with a monotonic occupational gradient. Additionally, we examined whether the observed disparities persist even after controlling for potential prognostic variables including clinicopathological features and smoking history.

## 2 MATERIALS AND METHODS

### 2.1 Data setting

We conducted a 5‐year overall survival analysis for bladder cancer patients diagnosed during 1970‐2011, using a population‐based data set (1970‐2016) of Kanagawa Cancer Registry (KCR), which covers the population of over nine million in Kanagawa Prefecture, representing 7% of the Japanese national population. Details of the study database have been previously described.^14^, ^15^ Briefly, Kanagawa Prefecture, a metropolitan prefecture located next to Tokyo, is the second largest prefecture in Japan, and KCR is one of the largest population‐based cancer registries in Japan. The data include basic information (sex, age, date of diagnosis, date of death/last follow‐up), and clinical information (pathology, stage, treatment). Additionally, KCR partly collected occupational and smoking history at diagnosis among the bladder cancer patients during 1970‐2011. However, on average, only 15% of the annually registered bladder cancer patients completed occupational information, and 19% completed smoking information; these data were no longer collected after 2016 due to the change of data management practice.^14^ KCR automatically updates dates of death/last follow‐up with population registers and death certificates, and previous diagnostic codes are updated to be consistent with changes in coding practice.^14^, ^15^ The occupational distribution in KCR parallels the national statistics as well as previous studies in Japan.^13^, ^14^, ^16^, ^17^, ^18^ We obtained a de‐identified data set under the research agreement between the authors and KCR, and the research ethics committees of The University of Tokyo, Tokyo (Protocol Number 3891‐4), and Kanto Rosai Hospital, Kanagawa (Protocol Number 2014‐38) approved the study.

### 2.2 Main outcome and study subjects

The main outcome was overall survival, defined by the person‐years from the date of initial bladder cancer diagnosis to the date of death/last follow‐up.

From a total of 23 906 bladder cancer patients registered in KCR with a diagnosis of incident bladder cancer (C67 in International Classification of Diseases, 10th revision) between 1970 and 2011, we excluded those with missing data for occupational information (20 313 patients, 85.0%), yielding an analytic sample of 3593 bladder cancer patients who had complete occupational information for analysis. The geographical locations of the study subjects varied from urbanized to rural areas. The occupational distribution of the analytic samples paralleled the national statistics as well as previous studies in Japan.^13^, ^14^, ^16^, ^17^, ^18^

### 2.3 Occupational class

From the longest‐held occupation at incident bladder cancer diagnosis listed in KCR based on the Japan Standard Occupational Classification, we identified major occupational categories for each patient as follows^13^, ^14^, ^16^, ^17^, ^18^: (a) professional and managerial workers, (b) clerical workers, (c) sales and service workers (including security, cleaning, carrying, and packaging workers), (d) agriculture, forestry, and fishery workers, (e) transportation workers (including machine operation workers), (f) construction and mining workers, (g) manufacturing workers, and (h) those not actively engaged in paid employment (eg, homemakers, students, unemployed, miscellaneous workers).

### 2.4 Covariates

**Figure 1:**
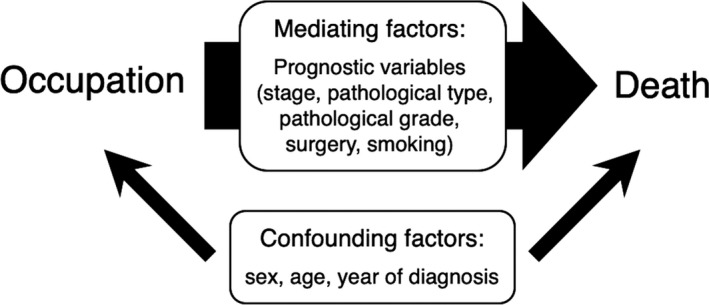
Confounding and mediating variables in the analytic model

Covariates included basic characteristics (sex, age, and year of diagnosis) as confounding factors (Figure 1). We adjusted for year of diagnosis as a continuous variable to control for potential secular changes in treatment regimens.^14^ Additionally, in a supplemental analysis, known prognostic factors were included in the regression analyses as potential mediating variables that may *explain* occupational disparities of bladder cancer survival (Figure 1)^10^, ^15^: summary stage (localized [early stage] vs regional invasion and distant metastasis [late stage]), pathological type (identified by International Classification of Disease for Oncology, Third edition pathological codes; urothelial carcinoma [8120‐8131 and 8050] vs non‐urothelial carcinoma), pathological grade (grade 3 or 4 [high‐grade] vs grade 1 or 2 [low‐grade]), surgery (yes/no), and smoking behaviors (never/ever). Due to the limitation in the data availability of the Union for International Cancer Control TNM staging information, we defined early (0, I) and late (II‐IV) stages in the subgroup analysis of bladder cancer patients after 2003.^14^, ^15^

### 2.5 Statistical analysis

The 5‐year overall survival rates were estimated by the Kaplan‐Meier curves and compared by logrank test. In our main analytic model (model 1), among the 3593 bladder cancer patients who completed occupational information, hazard ratios (HRs) and 95% confidence intervals (CIs) for overall death were estimated by Cox proportional hazard model, minimally adjusted for basic characteristics (sex, age, and year of diagnosis). Clerical workers served as the reference group for all analyses. For sensitivity analyses, to improve the completing rate on occupational information (15%), we performed subgroup analyses among (a) all male patients (n = 3278), the completing rate was 18% (3278 out of all 18 272 male bladder cancer patients during the study period) and (b) male patients aged < 70 (n = 1900), the completing rate was 24% (1900 out of all 7961 male bladder cancer patients aged < 70 during the study period). Additionally, we restricted analyses to a cohort of 826 bladder cancer patients diagnosed after 2003 with TNM staging information.

In a supplementary analysis to explain observed occupational differences in bladder cancer survival, we maximally adjusted for stage, pathology, treatment, and smoking behaviors. However, in this regression analysis among the 3593 study subjects, records included a large number of missing data: 88.1% (3165 patients) of stage information, 12.5% (448 patients) of pathological type, 79.2% (2846 patients) of pathological grade, 1.6% (57 patients) of treatment, and 72.3% (2596 patients) of smoking behaviors. We conducted multiple imputation for missing data among the 3593 study subjects with all variables used for analysis, and 20 imputed data sets were generated.^14^ Additionally, we estimated HRs and 95% CIs with multiple imputation among all 23 906 bladder cancer patients registered in KCR in the study period.

Alpha was set at 0.05, and all *P*‐values were two‐sided. Data were analyzed using STATA/MP13.1 (StataCorp LP).

## 3 RESULTS

**Figure 2:**
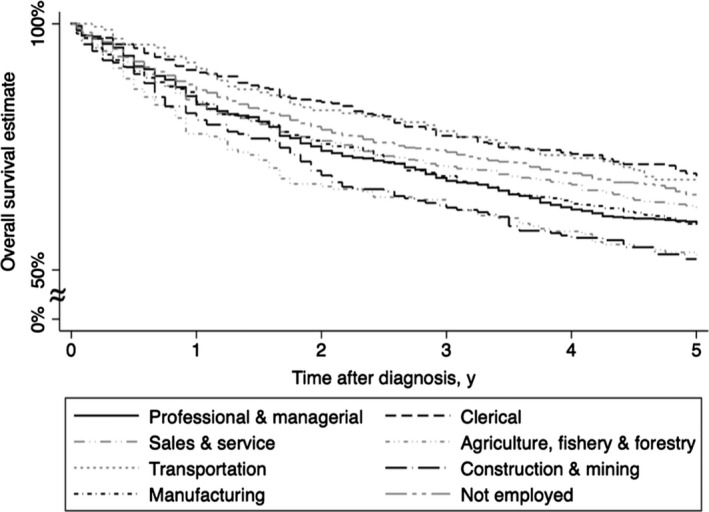
Overall survival curves by longest‐held occupations

**Figure 3:**
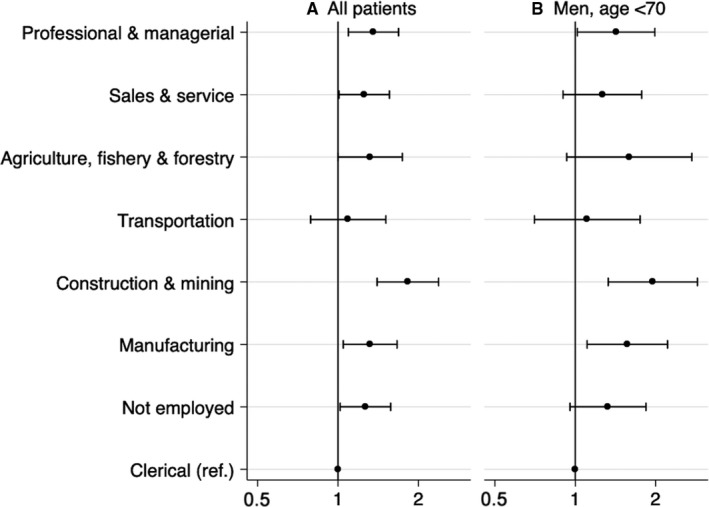
Occupational disparities in bladder cancer survival estimated with Cox proportional hazard model. Hazard ratios (circle) and 95% confidence intervals (line) were adjusted for sex, age, and year of diagnosis among (A) all study patients (n = 3593) and (B) male patients aged < 70 (n = 1900)

During the study period, the 5‐year overall survival was 61.9% (Figure 2 and Table 1). Significantly poorer prognoses were observed in professional and managerial workers (HR 1.36; 95% CI 1.09‐1.69), sales and service workers (HR 1.25, 95% CI 1.01‐1.56), construction and mining workers (HR 1.83, 95% CI 1.40‐2.38), manufacturing workers (HR 1.32, 95% CI 1.05‐1.66), and those not actively employed (HR 1.27, 95% CI 1.02‐1.58) compared with clerical workers (Figure 3 and Table 2). A poorer prognosis tended to be observed in agriculture, fishery, and forestry workers (HR 1.32, 95% CI 1.00‐1.74) compared with clerical workers, while prognosis in transportation workers did not differ from clerical workers (Figure 3 and Table 2). The sensitivity analyses with different subgroups of bladder cancer patients showed the similar pattern (Figure 3 and Table 2).

**Table 1:** Characteristics of bladder cancer patients who completed occupational information in Kanagawa Cancer Registry

Characteristics	Mean (SD) or number (%)		
	All patients, n = 3593	Men, n = 3278	Men, age < 70, n = 1900
Incidence rate, person‐year	0.10	0.10	0.08
5‐y survival estimate, %	61.9%	61.7%	69.7%
Women	315 (8.8%)	0 (0.0%)	0 (0.0%)
Age, y	67 (11)	67 (11)	59 (8)
Year of diagnosis	1995 (9)	1995 (9)	1994 (9)
Longest‐held occupation			
Professional & managerial	664 (18.5%)	601 (18.3%)	335 (17.6%)
Clerical	387 (10.8%)	345 (10.5%)	216 (11.4%)
Sales & service	725 (20.2%)	593 (18.1%)	343 (18.1%)
Agriculture, fishery, and forestry	189 (5.3%)	176 (5.4%)	49 (2.6%)
Transportation	168 (4.7%)	166 (5.1%)	113 (5.9%)
Construction and mining	218 (6.1%)	213 (6.5%)	135 (7.1%)
Manufacturing	449 (12.5%)	423 (12.9%)	241 (12.7%)
Not employed	793 (22.1%)	761 (23.2%)	468 (24.6%)
Stage	n = 428	n = 388	n = 191
Late‐stage	54 (12.6%)	50 (12.9%)	26 (13.6%)
Histological type	n = 3145	n = 2870	n = 1669
Non‐urothelial carcinoma	190 (6.0%)	159 (5.5%)	96 (5.8%)
Pathological grade	n = 747	n = 672	n = 370
High‐grade	242 (32.4%)	221 (32.9%)	127 (34.3%)
Treatment	n = 3536	n = 3228	n = 1874
Any surgery	3283 (92.8%)	2998 (92.9%)	1773 (94.6%)
Smoking behavior	n = 997	n = 910	n = 539
Ever smoker	670 (67.2%)	643 (70.7%)	402 (74.6%)

**Table 2:** Results of Cox proportional hazard model among bladder cancer patients with complete occupational information

Characteristics	Hazard ratio (95% confidence interval)			
	1970‐2016			2003‐2016
	All patients, n = 3593	Men, n = 3278	Men, age < 70, n = 1900	All patients, n = 826
Longest‐held occupation				
Clerical	1.00	1.00	1.00	1.00
Professional and managerial	1.36 (1.09, 1.69)**	1.42 (1.13, 1.79)**	1.42 (1.02, 1.99)*	1.52 (0.86, 2.72)
Sales & service	1.25 (1.01, 1.56)*	1.30 (1.03, 1.64)*	1.26 (0.90, 1.77)	1.06 (0.58, 1.93)
Agriculture, fishery, and forestry	1.32 (1.00, 1.74)	1.35 (1.01, 1.80)*	1.59 (0.93, 2.74)	2.31 (1.06, 5.07)*
Transportation	1.09 (0.79, 1.51)	1.11 (0.79, 1.55)	1.11 (0.70, 1.75)	1.27 (0.54, 3.00)
Construction and mining	1.83 (1.40, 2.38)***	1.93 (1.47, 2.53)***	1.95 (1.33, 2.87)***	2.89 (1.48, 5.62)**
Manufacturing	1.32 (1.05, 1.66)*	1.31 (1.03, 1.67)*	1.57 (1.11, 2.22)*	1.88 (1.02, 3.47)*
Not employed	1.27 (1.02, 1.58)*	1.31 (1.04, 1.65)*	1.33 (0.96, 1.84)	1.16 (0.66, 2.04)
Women	1.02 (0.84, 1.24)	NA	NA	0.82 (0.51, 1.30)
Age	1.04 (1.03, 1.05)***	1.04 (1.03, 1.05)***	1.02 (1.01, 1.03)**	1.06 (1.05, 1.07)***
Year of diagnosis	0.98 (0.97, 0.99)***	0.98 (0.97, 0.98)***	0.98 (0.97, 0.99)***	0.93 (0.88, 0.99)*

* *P* < .05.

** *P* < .01.

*** *P* < .001.

In a supplementary analysis, although the observed occupational difference was partly attenuated after adjustment for prognostic variables, the occupational disparities remained significant for professional and managerial workers and construction and mining workers (Table 3). The pattern was mostly similar among all bladder cancer patients registered in KCR in the study period (Table 3).

**Table 3:** Cox proportional hazard model with multiple imputation among bladder cancer patients in Kanagawa Cancer Registry

Characteristics	Hazard ratio (95% confidence interval)		
	Complete occupational information^a^		All bladder cancer patients^b^
	All patients (1970‐2016) n = 3593	Subgroup with TNM staging (2003‐2016) n = 826	All patients (1970‐2016) n = 23 906
Longest‐held occupation			
Clerical	1.00	1.00	1.00 (1.00, 1.00)
Professional and managerial	1.27 (1.01, 1.60)*	1.37 (0.75, 2.49)	1.19 (1.00, 1.41)*
Sales and service	1.15 (0.91, 1.44)	0.96 (0.50, 1.82)	1.17 (0.98, 1.39)
Agriculture, fishery, and forestry	1.30 (0.97, 1.73)	1.75 (0.76, 4.02)	1.26 (1.00, 1.58)*
Transportation	1.05 (0.75, 1.47)	1.10 (0.43, 2.80)	1.17 (0.91, 1.51)
Construction and mining	1.65 (1.21, 2.24)**	2.39 (1.20, 4.77)*	1.44 (1.16, 1.78)**
Manufacturing	1.25 (0.98, 1.60)	1.36 (0.70, 2.62)	1.24 (1.04, 1.49)*
Not employed	1.17 (0.91, 1.51)	0.96 (0.53, 1.72)	1.23 (1.00, 1.52)
Women	1.03 (0.78, 1.37)	0.75 (0.45, 1.24)	1.11 (1.02, 1.21)*
Age	1.03 (1.02, 1.04)***	1.06 (1.05, 1.08)***	1.05 (1.05, 1.05)***
Year of diagnosis	0.99 (0.97, 1.02)	0.97 (0.90, 1.04)	0.99 (0.97, 1.00)
Late stage	2.26 (0.84, 6.04)	2.94 (1.89, 4.58)***	2.53 (1.67, 3.84)***
Non‐urothelial carcinoma	1.97 (1.46, 2.66)***	1.55 (0.90, 2.69)	1.61 (1.41, 1.84)***
High‐grade	1.61 (1.21, 2.16)**	1.58 (1.00, 2.50)	1.35 (1.18, 1.54)***
Any surgery	0.42 (0.30, 0.59)***	0.61 (0.38, 0.97)*	0.81 (0.69, 0.95)*
Ever smoker	1.03 (0.72, 1.47)	1.09 (0.78, 1.51)	1.02 (0.92, 1.13)

a Missing data for stage, pathological type and grade, surgery, and smoking were multiply imputed.

b Missing data for occupation, stage, pathological type and grade, surgery, and smoking were multiply imputed.

* *P* < .05.

** *P* < .01.

*** *P* < .001.

## 4 DISCUSSION

As far as we are aware, our study is the first to demonstrate occupational disparities in bladder cancer survival in Japan. Contrary to expectation, we did not find a monotonic gradient in survival according to occupation, that is, professional and managerial workers experiencing the most favorable survival chances. Instead, we found that compared with clerical workers, the 5‐year overall survival was worse among professional and managerial workers, as well as among construction, sales and service, and manufacturing workers, and those not actively employed. Although potential occupational disparities in prognostic factors (clinical and pathological features and smoking habits) have been thought to underlie occupational disparities in bladder cancer survival in previous studies,^8^, ^9^ the occupational disparity remained significant even after controlling for relevant prognostic factors in the current study. Therefore, other pathways not included in conventional clinicopathological prognostic factors may have played a role.

For example, physically active patients tend to have better cancer prognosis for cancers of the breast, colorectum, and prostate compared with their sedentary counterparts.^19^ Although the benefits of active lifestyle have not been documented on bladder cancer survival, sedentary lifestyle behaviors and overweight/obesity were associated with bladder cancer risk and overweight/obesity was associated with increased risk of cancer recurrence and progression.^19^, ^20^, ^21^, ^22^ In Japan, the highest level of leisure‐time physical activity tends to be observed in clerical workers, while the lowest level tends to be observed in white‐collar workers (including professional and managerial workers) and blue‐collar workers (including construction and manufacturing workers).^23^

Workplace environmental factors may partly explain the poorer prognosis in blue‐collar occupations, particularly in construction and mining workers. Workers in construction and mining industries are likely to be exposed to dusty air and chemical hazards, and as a result may experience worse prognosis for not only bladder cancer but also major cancer sites, including lung, stomach, and colorectal cancers.^10^, ^12^ In the current study, each patient's longest‐held occupation was used as an indicator of socioeconomic status, and was not designed to capture specific occupational/environment exposure to carcinogens. However, construction and mining workers had the poorest survival in bladder cancer, which is consistent with previous findings.^10^

Psychological pathways, including job stress, may also partly explain our results. Poor mental health conditions are associated with worse bladder cancer prognosis,^24^ and high job stress tended to be seen among not only blue‐collar workers but also white‐collar workers in Japan, which contrasts with the pattern seen in Western countries.^13^, ^18^, ^25^ Chronic job stress may trigger systemic inflammation and stimulate the immune system, which is reflected by increased levels of white blood cell counts.^26^ Besides, neutrophil‐to‐lymphocyte ratio in differential leukocyte counts is a biomarker of systematic inflammation response, and systematic reviews and meta‐analyses suggest that a poorer bladder cancer prognosis is associated with higher levels of neutrophil‐to‐lymphocyte ratio.^27^, ^28^, ^29^, ^30^ Therefore, as seen in recent studies for other cancer mortalities in Japan,^11^, ^12^ it is plausible that bladder cancer patients in blue‐collar and the highest class occupations (ie, professionals and managers) might have a worse prognosis compared with clerical workers.

A further potential behavioral mechanism for occupational disparities in bladder cancer survival is timely receipt of treatment, a key factor in bladder cancer prognosis. Japan introduced a universal health care system in 1961, and access to bladder cancer treatment is available to patients irrespective of their socioeconomic status, which should have flattened the occupational gradient in bladder cancer survival.^31^ However, as suggested previously, socially disadvantaged groups may have a higher likelihood of delaying the initiation of treatment, which may result in poor prognosis in that group.^32^

Additionally, the occupational disparities in smoking behavior could partly explain the residual disparities in bladder cancer survival. In Japan, higher occupational class workers tend to smoke as much (or sometimes even more) compared with their lower occupational class counterparts, and the occupational distribution of smoking behaviors differs markedly from Western countries.^13^, ^33^ Therefore, it would be plausible that working in the highest occupational classes did not show the most favorable survival chance in the current study.

Several limitations should be noted. First, although our data set was based on a population‐based cancer registry, our internal validity and external generalizability were limited due to sizable missing data. Additionally, self‐employed workers (eg, own a small family business) might be potentially misclassified to a high‐status occupational class (eg, managerial positions in a huge industrial company). Although the observed occupational disparities did not materially change in sensitivity analyses among different subgroups using multiple imputation, the results were based on imputed data from the 15% of bladder cancer patients registered in KCR. However, occupational distribution in KCR parallels the national statistics and previous studies in Japan.^13^, ^14^, ^16^, ^17^, ^18^ Second, although studies suggest that occupational class is associated with educational attainment in Japan,^11^ we could not assess the contribution of relevant socioeconomic indicators (ie, educational attainment and income), physical activity, obesity, job stress, neutrophil‐to‐lymphocyte ratio, detailed smoking behavior (status and intensity), as well as timely standardized treatments and treatment regimens.^34^ Despite these limitations, although previous studies did not sufficiently assess possible known prognostic factors,^8^, ^9^ we controlled for those prognostic factors for a subset of our patients.

In conclusion, occupational disparities in bladder cancer survival appeared to exist in Japan even after controlling for known prognostic factors, suggesting occupation may be a crucial independent determinant of bladder cancer survival. However, questions remain regarding whether the major risk behavior of smoking and other potential psychological and behavioral pathways may explain the residual occupational disparities. Hence, future studies should attempt to integrate all of the clinicopathological, psychological, and behavioral aspects, in order to overcome occupation‐oriented survival inequalities in this site.

## CONFLICT OF INTEREST

The authors declare no potential conflict of interest.

## AUTHOR CONTRIBUTIONS

Conception and design, Masayoshi Zaitsu, Ichiro Kawachi: Development of methodology, Masayoshi Zaitsu, Ichiro Kawachi: Acquisition of data, Masayoshi Zaitsu, Takumi Takeuchi: Analysis and interpretation of data, Masayoshi Zaitsu, Hye‐Eun Lee, Sangchul Lee, Takumi Takeuchi, Yasuki Kobayashi, Ichiro Kawachi: Writing, review and/or revision of the manuscript, Masayoshi Zaitsu, Hye‐Eun Lee, Sangchul Lee, Takumi Takeuchi, Yasuki Kobayashi, Ichiro Kawachi: Administrative, technical, or material support, Masayoshi Zaitsu, Takumi Takeuchi, Yasuki Kobayashi, Ichiro Kawachi: Study supervision, Masayoshi Zaitsu, Takumi Takeuchi, Yasuki Kobayashi, Ichiro Kawachi.

## Data Availability

The data that support the findings of this study are available from the Kanagawa Cancer Registry (KCR). Restrictions apply to the availability of these data, which were used under license for this study by the KCR; research data used in the study cannot be made publicly available directly by the authors. If any person wishes to verify our data analysis, they are most welcome to contact the corresponding author.

## References

[1] Antoni S , Ferlay J , Soerjomataram I , Znaor A , Jemal A , Bray F . Bladder cancer incidence and mortality: a global overview and recent trends. Eur Urol. 2017;71(1):96‐108. 27370177 10.1016/j.eururo.2016.06.010

[2] Cumberbatch MG , Cox A , Teare D , Catto JW . Contemporary occupational carcinogen exposure and bladder cancer: a systematic review and meta‐analysis. JAMA Oncol. 2015;1(9):1282‐1290. 26448641 10.1001/jamaoncol.2015.3209

[3] Freedman ND , Silverman DT , Hollenbeck AR , Schatzkin A , Abnet CC . Association between smoking and risk of bladder cancer among men and women. JAMA. 2011;306(7):737‐745. 21846855 10.1001/jama.2011.1142 PMC3441175

[4] Hadkhale K , MacLeod J , Demers PA , et al. Occupational variation in incidence of bladder cancer: a comparison of population‐representative cohorts from Nordic countries and Canada. BMJ Open. 2017;7(8):e016538. 10.1136/bmjopen-2017-016538 PMC5629726 28780557

[5] Hadkhale K , Martinsen JI , Weiderpass E , et al. Occupation and risk of bladder cancer in nordic countries. J Occup Environ Med. 2016;58(8):e301‐e307. 27294445 10.1097/JOM.0000000000000803

[6] Cumberbatch MG , Windsor‐Shellard B , Catto JW . The contemporary landscape of occupational bladder cancer within the United Kingdom: a meta‐analysis of risks over the last 80 years. BJU Int. 2017;119(1):100‐109. 27332981 10.1111/bju.13561

[7] Nakano M , Omae K , Takebayashi T , Tanaka S , Koda S . An epidemic of bladder cancer: ten cases of bladder cancer in male Japanese workers exposed to ortho‐toluidine. J Occup Health. 2018;60(4):307‐311. 29743389 10.1539/joh.2017-0220-OA PMC6078838

[8] Auvinen A , Karjalainen S , Pukkala E . Social class and cancer patient survival in Finland. Am J Epidemiol. 1995;142(10):1089‐1102. 7485054 10.1093/oxfordjournals.aje.a117562

[9] Noon AP , Martinsen JI , Catto JWF , Pukkala E . Occupation and bladder cancer phenotype: identification of workplace patterns that increase the risk of advanced disease beyond overall incidence. Eur Urol Focus. 2018;4(5):725‐730. 28753772 10.1016/j.euf.2016.06.014

[10] Smith ND , Prasad SM , Patel AR , et al. Bladder cancer mortality in the United States: a geographic and temporal analysis of socioeconomic and environmental factors. J Urol. 2016;195(2):290‐296. 26235377 10.1016/j.juro.2015.07.091

[11] Tanaka H , Nusselder WJ , Bopp M , et al. Mortality inequalities by occupational class among men in Japan, South Korea and eight European countries: a national register‐based study, 1990–2015. J Epidemiol Community Health. 2019;73(8):750‐758. 31142611 10.1136/jech-2018-211715 PMC6678055

[12] Eguchi H , Wada K , Prieto‐Merino D , Smith DR . Lung, gastric and colorectal cancer mortality by occupation and industry among working‐aged men in japan. Sci Rep. 2017;7:43204. 28230191 10.1038/srep43204 PMC5322319

[13] Zaitsu M , Kato S , Kim Y , et al. Occupational class and risk of cardiovascular disease incidence in Japan: nationwide, multicenter, hospital‐based case‐control study. J Am Heart Assoc. 2019;8(6):e011350. 30845875 10.1161/JAHA.118.011350 PMC6475056

[14] Zaitsu M , Kim Y , Lee HE , Takeuchi T , Kobayashi Y , Kawachi I . Occupational class differences in pancreatic cancer survival: a population‐based cancer registry‐based study in Japan. Cancer Med. 2019;8(6):3261‐3268. 30953422 10.1002/cam4.2138 PMC6558482

[15] Zaitsu M , Toyokawa S , Tonooka A , et al. Sex differences in bladder cancer pathology and survival: analysis of a population‐based cancer registry. Cancer Med. 2015;4(3):363‐370. 25533611 10.1002/cam4.379 PMC4380962

[16] Zaitsu M , Kaneko R , Takeuchi T , Sato Y , Kobayashi Y , Kawachi I . Occupational class and male cancer incidence: nationwide, multicenter, hospital‐based case‐control study in Japan. Cancer Med. 2019;8(2):795‐813. 30609296 10.1002/cam4.1945 PMC6382925

[17] Zaitsu M , Kaneko R , Takeuchi T , Sato Y , Kobayashi Y , Kawachi I . Occupational inequalities in female cancer incidence in Japan: hospital‐based matched case‐control study with occupational class. SSM Popul Health. 2018;8(5):129‐137. 10.1016/j.ssmph.2018.06.001 PMC6019265 30014030

[18] Zaitsu M , Cuevas AG , Trudel‐Fitzgerald C , Takeuchi T , Kobayashi Y , Kawachi I . Occupational class and risk of renal cell cancer. Health Sci Rep. 2018;1(6):e49. 30623081 10.1002/hsr2.49 PMC6266576

[19] Mctiernan A , Friedenreich CM , Katzmarzyk PT , et al. Physical activity in cancer prevention and survival: a systematic review. Med Sci Sports Exerc. 2019;51(6):1252‐1261. 31095082 10.1249/MSS.0000000000001937 PMC6527123

[20] Keimling M , Behrens G , Schmid D , Jochem C , Leitzmann MF . The association between physical activity and bladder cancer: systematic review and meta‐analysis. Br J Cancer. 2014;110(7):1862‐1870. 24594995 10.1038/bjc.2014.77 PMC3974090

[21] Noguchi JL , Liss MA , Obesity PJK . Physical activity and bladder cancer. Curr Urol Rep. 2015;16(10):74,015–0546‐2. 10.1007/s11934-015-0546-2 26303776

[22] Westhoff E , Witjes JA , Fleshner NE , et al. Body mass index, diet‐related factors, and bladder cancer prognosis: a systematic review and meta‐analysis. Bladder Cancer. 2018;4(1):91‐112. 29430510 10.3233/BLC-170147 PMC5798521

[23] Takao S , Kawakami N , Ohtsu T ; Japan Work Stress and Health Cohort Study Group . Occupational class and physical activity among Japanese employees. Soc Sci Med. 2003;57(12):2281‐2289. 14572837 10.1016/s0277-9536(03)00134-5

[24] Pham H , Torres H , Sharma P . Mental health implications in bladder cancer patients: a review. Urol Oncol. 2019;37(2):97‐107. 30584034 10.1016/j.urolonc.2018.12.006

[25] Kawakami N , Haratani T , Kobayashi F , et al. Occupational class and exposure to job stressors among employed men and women in Japan. J Epidemiol. 2004;14(6):204‐211. 15617394 10.2188/jea.14.204 PMC8784243

[26] Magnusson Hanson LL , Westerlund H , Goldberg M , et al. Work stress, anthropometry, lung function, blood pressure, and blood‐based biomarkers: a cross‐sectional study of 43,593 French men and women. Sci Rep. 2017;7(1):9282. 28839130 10.1038/s41598-017-07508-x PMC5570902

[27] Li X , Ma X , Tang LU , et al. Prognostic value of neutrophil‐to‐lymphocyte ratio in urothelial carcinoma of the upper urinary tract and bladder: a systematic review and meta‐analysis. Oncotarget. 2016;8(37):62681‐62692. 28977980 10.18632/oncotarget.17467 PMC5617540

[28] Lucca I , Jichlinski P , Shariat SF , et al. The Neutrophil‐to‐lymphocyte ratio as a prognostic factor for patients with urothelial carcinoma of the bladder following radical cystectomy: validation and meta‐analysis. Eur Urol Focus. 2016;2(1):79‐85. 28723455 10.1016/j.euf.2015.03.001

[29] Tang X , Du P , Yang Y . The clinical use of neutrophil‐to‐lymphocyte ratio in bladder cancer patients: a systematic review and meta‐analysis. Int J Clin Oncol. 2017;22(5):817‐825. 28752351 10.1007/s10147-017-1171-5

[30] Vartolomei MD , Porav‐Hodade D , Ferro M , et al. Prognostic role of pretreatment neutrophil‐to‐lymphocyte ratio (NLR) in patients with non‐muscle‐invasive bladder cancer (NMIBC): a systematic review and meta‐analysis. Urol Oncol. 2018;36(9):389‐399. 29884342 10.1016/j.urolonc.2018.05.014

[31] Schinkel JK , Shao S , Zahm SH , McGlynn KA , Shriver CD , Zhu K . Overall and recurrence‐free survival among black and white bladder cancer patients in an equal‐access health system. Cancer Epidemiol. 2016;42:154‐158. 27161431 10.1016/j.canep.2016.04.012 PMC5727912

[32] Gray PJ , Fedewa SA , Shipley WU , et al. Use of potentially curative therapies for muscle‐invasive bladder cancer in the United States: results from the National Cancer Data Base. Eur Urol. 2013;63(5):823‐829. 23200811 10.1016/j.eururo.2012.11.015

[33] Lahelma E , Pietiläinen O , Ferrie J , et al. Changes over time in absolute and relative socioeconomic differences in smoking: a comparison of cohort studies from Britain, Finland, and Japan. Nicotine Tob Res. 2016;18(8):1697‐1704. 26764256 10.1093/ntr/ntw004 PMC4941597

[34] Tomaszewski JJ , Handorf E , Corcoran AT , et al. Care transitions between hospitals are associated with treatment delay for patients with muscle invasive bladder cancer. J Urol. 2014;192(5):1349‐1354. 24835054 10.1016/j.juro.2014.05.027 PMC4422495

